# Phenotypic and Immunometabolic Aspects on Stem Cell Memory and Resident Memory CD8^+^ T Cells

**DOI:** 10.3389/fimmu.2022.884148

**Published:** 2022-06-17

**Authors:** Marco Pio La Manna, Mojtaba Shekarkar Azgomi, Bartolo Tamburini, Giusto Davide Badami, Leila Mohammadnezhad, Francesco Dieli, Nadia Caccamo

**Affiliations:** ^1^ Central Laboratory of Advanced Diagnosis and Biomedical Research (CLADIBIOR) Azienda Ospedaliera Universitaria Policlinico (A.O.U.P.) Paolo Giaccone, University of Palermo, Palermo, Italy; ^2^ Department of Biomedicine, Neurosciences and Advanced Diagnostic (Bi.N.D.), University of Palermo, Palermo, Italy

**Keywords:** CD8 T_RM_ cells, CD8 T_SCM_ cells, metabolism, differentiation, infectious diseases

## Abstract

The immune system, smartly and surprisingly, saves the exposure of a particular pathogen in its memory and reacts to the pathogen very rapidly, preventing serious diseases.

Immunologists have long been fascinated by understanding the ability to recall and respond faster and more vigorously to a pathogen, known as “memory”.

T-cell populations can be better described by using more sophisticated techniques to define phenotype, transcriptional and epigenetic signatures and metabolic pathways (single-cell resolution), which uncovered the heterogeneity of the memory T-compartment. Phenotype, effector functions, maintenance, and metabolic pathways help identify these different subsets. Here, we examine recent developments in the characterization of the heterogeneity of the memory T cell compartment. In particular, we focus on the emerging role of CD8^+^ T_RM_ and T_SCM_ cells, providing evidence on how their immunometabolism or modulation can play a vital role in their generation and maintenance in chronic conditions such as infections or autoimmune diseases.

## Introduction

T lymphocytes are an essential component of adaptive immunity known for their role in protecting against infectious diseases caused by invasive pathogens and cancer cells but also for their crucial role as important target cells in immunotherapy (1–5).

Memory T cells differ in their naïve counterparts for several functional characteristics identified in booster reactions due to a second encounter with the pathogen. These T memory populations are organized in a developmental hierarchy, where stem cell memory T-cells (T_SCM_) renew themselves and generate long-lived central memory T-cells (T_CM_) and short-lived memory T-cells (T_EM_) (6–10). However, the mechanisms underlying the increased multipotency of T_SCM_ cells relative to T_CM_ cells were not clearly defined in the molecular terms (11).

The principal characteristics of the memory T-cell responses include several aspects such as an increased pool of specific memory T cells thanks to the recognition of pathogen-derived-antigens by TCR, a faster and more potent response to the infection; a pre-programming to generate a “tailor-made” set of effector cell types optimized to combat pathogens, which includes the role of T follicular helper cell (T_FH_) responses involved in helping humoral immunity; the presence of memory T cells in barrier tissues as sentinels in detecting and fighting infection. This last characteristic is dominated by resident memory T cells (T_RM_) that are maintained in non-lymphoid tissues and do not exchange with populations in the circulation. Since this population is not represented in the blood, it is difficult to evaluate its generation and persistence.

Once T cells are not properly primed or generated, they gradually lose their ability to resolve the chronic infection. Such T-cell memory populations are likely to disappear over time or become permanently dysfunctional, favoring the persistence of the pathogen. As a result, T lymphocyte responses can become dangerous, resulting in excessive tissue damage, collectively referred to as immunopathology, which may determine the immune system’s persistent systemic alteration, affecting the development of memory cells and their response to the re-encounter with the same pathogen.

The aim of this review is to focus on the role of T_SCM_ and T_RM_ cell subsets in the course of infectious and autoimmune diseases and how the metabolic program can impact the features of the immune response.

## The Basic Concept of CD8^+^ T Cell Memory Subsets

The major T cell subsets are classified into CD4^+^ T and CD8^+^ T cells based on cell functions and cell surface markers. CD4^+^ T cells, also known as T helper (Th) cells, play an essential role in the adaptive immune response to pathogens due to their multiple roles in immune response orchestration (12), such as helping B cells to produce antibodies, recruiting granulocytes to infected sites, and producing cytokines and chemokines. Many distinct CD4^+^ T cell subsets have been identified since the identification of Th1 by *Mosmann et al.* (12–15). CD8^+^ T cells are essential for immune defense against intracellular pathogens (16, 17) and tumoral cells.

For several years, memory T cells were divided into two main principal subsets: central memory T cells (T_CM_) and effector memory T cells (T_EM_) (18). This initial classification was based on the expression of the lymphoid-homing molecules CCR7 and L-selectin (CD62L), while T_EM_ were identified on the bases of the reduced expression of these receptors relative to T naive (T_N_) and T_CM_ and for their presence as recirculating cells between the blood and peripheral tissues. After this first characterization of the different subsets, additional surface markers have been added, such as the mutually exclusive expression of the two isoforms of the CD45R molecule, CD45RA or CD45RO. In different species, such as mice, T_EM_ has been shown to retain lower expression of molecules involved in long-term persistence (19), human primates (20, 21) and humans (22, 23), while upregulating transcription factors (TFs) mediating terminal differentiation with decreased self-renewal and multipotent capacity compared with T_CM_ (7).

Furthermore, T_EM_ cells have limited expansion potential and are unable to enter the lymph nodes from the blood, but are marked by the expression of genes associated with cytotoxicity and can perform rapid effector functions when renewing TCR signalling (24). For a long time, T_EM_ cells were thought to be superior for penetrating and examining peripheral tissues; however, recent research indicates that T_EM_ cells and T_EM_-like cells are largely excluded from human and mouse non-lymphoid tissues (NLTs) (23, 25–27).

T_CM_ cells can be distinguished by the high-level expression of CD62L and CCR7 lymphoid localization markers and their capacity to recirculate between blood and lymphoid tissues. They are considered to be multipotent, and at least one subset of this cell pool shows increased expansion potential when the antigen is encountered (8). Two novel memory subsets have emerged during the past decade, further highlighting the inherent diversity among these subtypes. In particular, a noncirculating lineage of T cells, the T_RM_, express tissue-residency markers, CD69 and CD103, and reside in peripheral tissues (28–30). This subset is located at the entry point of invading pathogens to provide the first rapid defense line (28–30). While, T_SCM_, defined in humans by surface expression of CD122, CD95, and CXCR3 within the CD45RA^+^CD45RO^–^CCR7^+^CD62L^+^ naive-like compartment, are characterized by increased homeostatic capacity for automatic renewal, increased proliferation capacity, and multipotent development potential compared to T_CM_ and T_EM_; moreover, this subset is capable of generating differentiated *in vitro* in cells, including T_CM_ and T_EM_ or as adoptive transfer in xenogeneic mouse models, as well as in humans (8, 9, 20, 31–33). Although this subset was initially identified in mice as part of an allogeneic bone marrow transplantation (34) or induced by T naive (T_N_) activated by the inhibition of glycogen synthase kinase 3b (GSK-3b) (35).

Although T_SCM_ and T_CM_ have the ability to retain the expression of associated naive genes while simultaneously displaying the rapid production of cytokine upon stimulating T-cell receptor (TCR), the T memory subsets as a whole are often represented as having mutually exclusive properties, primarily a potential similar stem-like potential (T_SCM_ and T_CM_) versus immediacy of effector functions/cytotoxicity (T_EM_) (7, 8, 22, 36, 37).

An important aspect of CD8^+^ T cells is their ability to adapt their functions to the basic type of pathogens encountered. Firstly, the recognition of the antigen, T cells, through the clonal expansion process, become biased to recognize pathogens to which they have already been exposed (38). Moreover, remodelling of the epigenetic landscape enables the memory cells that form in this process to perform effective functions more rapidly (39). The distribution of the memory compartment of the CD8^+^ T cells at different body sites maximizes the chances of early recognition of pathogens during a renewed infection (40). In accordance with the concept that the CD8^+^ storage T-cell pool can provide fast effector functions and has the ability to renew clonal expansion, this cell pool is very diverse at epigenetic, transcriptional and protein expression levels.

**Figure 1:**
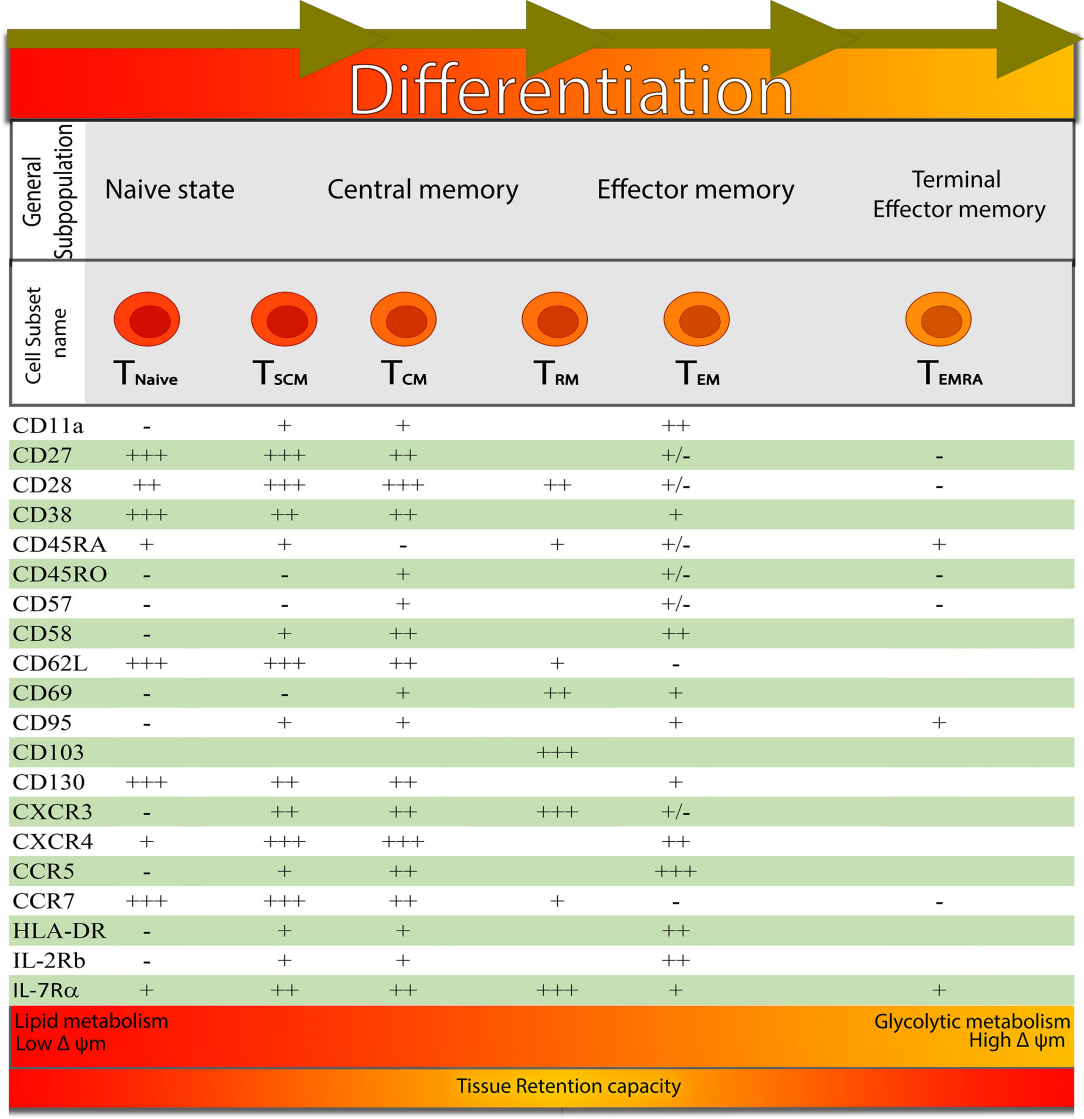
Signatures for distinguishing the different developmental T cell stages: from left to right, it shows the different development stages of CD8^+^ T cell-based on the expression of surface markers. At the bottom of the figure, the main metabolic pathway and mitochondrial membrane potential that are favoured at the functional and phenotypic stage are represented by different coloured gradients (from red to orange), indicating that T_RM_ cells have a larger tissue retention capacity.

It is believed that these memory populations are organized in a developmental hierarchy, according to which T_SCM_ renew themselves and generate a long-lasting T_CM_, T_EM_ cells, and T_RM_ cells (6–10) (**Figure 1**). However, the mechanisms underlying the improved multi-potency of T_SCM_ cells compared to T_CM_ cells have not been clearly defined in molecular terms (11).

In addition to the CD8^+^ systemic memory T-cell pool, one can distinguish a T_RM_ cell pool from CD8^+^ in tissues that permanently reside in non-lymphoid tissues (NLTs).

By a continuous migration and monitoring process that is limited to distinct anatomical compartments, such as stroma or organ parenchyma, T_RM_ cells patrol tissues to scan for foreign invaders (41, 42). After encountering the antigen, T_RM_ cells quickly induce a local alarm state, resulting in the recruitment of other immune cells and local production of antibacterial and antiviral proteins by epithelial cells (43, 44).

In keeping with this “pathogen alert” function, T_RM_ cells produce not only cytotoxic molecules, such as granzyme B and perforin but also cytokines such as IFN-γ and TNF, which may affect the behaviour of neighbouring cells (45–50). In addition, the existence of T_RM_ cells that express minimal levels of cytolytic molecules and therefore rely primarily on this pathogen, warning function, has been reported in several human tissues (51–54).

It has been proven that the differentiation of T cells from memory can be corrupted under conditions of persistent antigenic stimulation, this finding was primarily observed in chronic viral infections and progressive malignant tumours, which promote a state of T-cell exhaustion which is characterized by an organized loss of effective function, altered proliferation and increased regulation of inhibitor receptors (55). This dynamic process takes place over a period of weeks after initiation (56, 57) and involves a genome-wide accumulation of epigenetic changes (58, 59).

Recent studies have shown that exhausted T-cell (TEX) populations are developmental and functionally heterogeneous, incorporating stem-like progenitors that express T-cell factor 1 (TCF1), which produce highly differentiated TEX cells that are basically dysfunctional and do not have TCF1 (60–63). It is important to note that the therapeutic benefits of blocking immune checkpoints in the context of chronic viral infections and various cancers are believed to function *via* these TCF1^+^ progenitors, which appear to be susceptible to interventions that specifically target the inhibitory receptor programmed death-1 (PD-1) (60, 62, 64–67).Current data suggest that exhausted and functional memory T cells originate from distinct populations of stem-like progenitors engaged in distinct destinies. However, the precise nature of these stem-like progenitors, which shape the adaptive immune response and influence the outcome of many globally relevant diseases, has remained unclear. Very recently, Galletti et al. (68), with the aim of a complete and impartial approach, have mapped the origins of dysfunctional and functional CD8^+^ human memory T-cells. Their data allow the identification of two distinct subsets of CCR7^+^ progenitors in healthy individuals that are distinguished by the expression of PD-1 and TIGIT. They identified a progenitor committed the cells to generate a dysfunctional subset with an exhausted progeny expressing these two inhibitor receptors. In contrast, they identified another progenitor cell committed to generating a more functional progeny where the absence of these inhibitory receptors was observed. Therefore, the transcriptional assessment of the distinct subset PD-1^+^TIGIT^+^ also explained most of the differences between the T_SCM_ and T_CM_ cells, providing a clearer view of how human CD8^+^ memory T-cells differentiate.

Recent advances in single-cell technologies at the proteomic and transcriptomic levels, along with genome-scale epigenetic profiling studies, provided new information that is refining our current understanding of gene expression programs and the developmental origin of memory T cells. In particular, single-cell transcriptomic has identified subsets of human and murine T cells that simultaneously co-express stem and effector genes. In addition, the epigenetic profiling of stem T cells has made it possible to identify several locus effectors in these cells which remain epigenetic, while having limited transcriptional activity, explaining their ability to quickly recall an effective response (69, 70).

T cell memory has a unique set of functional properties and potentials that set it apart from many of its sisters. This is not to say that every T-cell in memory is its own subset; rather, it is to say that one phenotypic or functional feature does not imply another.

## Metabolic Regulation of Memory T-Cell Differentiation and Function

Very recently, the role of metabolism in the immune system has become more evident. Inactivated naive T cells migrate continuously under immune surveillance *via* secondary lymphoid organs at the expense of ATP and thus require basal replacement biosynthesis. Resting T cells are more dependent on energy-efficient processes such as the β-oxidation of fatty acids and the oxidation of pyruvate and glutamine *via* the tricarboxylic acid cycle (TCA cycle). To maintain this energy-providing basal metabolism, resting T cells need specific signals *via* TCR or cytokine receptors such as IL-7R (71). Furthermore, resting T cells do not have a static and well-determined metabolism but undergo dynamic regulation. For example, environmental signals can control the utilization of nutrients in resting T cells, causing them to become trophic and influencing the ability to initiate cell proliferation and resistance to apoptosis. IL-7R regulates glucose absorption mainly thanks to the PI3k-Akt-mTOR pathway, which promotes the trafficking of Glut1 on the cell surface (72, 73). As a result, IL-7R regulation of glycolysis is critical for T lymphocyte basal metabolism *in vivo*, as its deletion results in cell atrophy and the inability to maintain glycolysis *in vivo (* 74).

Unlike naive T lymphocytes, T memory (T_M_) lymphocytes usually divide every 2-3 weeks undergoing intermittent cell division. These lymphocytes necessarily require the combination of IL-15 and IL-7 for their proliferation and survival to which a high level of CD127 and CD122 molecules is associated, respectively regulated by FOX01 and by, T-bet and eomesodermin (17, 75).

Furthermore, T_M_ cells and their responses are dependent on lipid oxidation, and although the homeostasis of the various subgroups is very similar, there are important differences. In fact, CD4^+^ T_M_ cells appear to be less dependent on IL-15 signal than CD8^+^ T_M_ cells, and this is probably partly determined by the low expression of CD 122 on CD4^+^ T_M_ cells (76).

Homeostasis of memory T cells is determined by IL-15 and/or IL-7, which keep them in a precarious state of quiescence (G1 phase of the cell cycle). Aerobic glycolysis and mitochondrial metabolism have important and well-defined roles in the differentiation of T_M_ cells, but mitochondrial metabolism supports their quiescent, survival, and functions (77, 78). Increased mitochondrial function and acetate uptake mediate rapid recall responses by memory T cells, both in T_CM_ and T_RM_ cells. Thus, metabolism influences entry and exit from quiescence to regulate their generation, survival, and function (79, 80).

Although it is clear why the transition from quiescent and naive T cells to proliferating T cells results in a series of altered metabolic demands, it is less clear why differentiated active T cell pool memory cells necessitate a carefully orchestrated transition to invert the cell focus on vigorous expansion and equip emerging memory cells for sustained maintenance and the enhanced metabolic capacity required to mount a rapid recall response. Differentiating effectors involve a spectacular reprogramming of metabolic functions, where the requirements for increased biosynthesis and energy use are met in the most obvious way by induction aerobic glycolysis (oxidative phosphorylation, characteristic of the catabolic state of naive cells, is also maintained but plays a proportionately lesser role).

Cells need to reprogram their metabolism to leave rapidly expanding and reenter the memory pool (81). For the first time, it has been shown by pioneering studies that mTOR inhibition and enhanced fatty acid oxidation (FAO) induce an increase in CD8^+^ differentiated memory T cells (82–84).

Above all, memory cells’ metabolic status differs from that of naive T cells; changes in mitochondrial mass and FAO capacity (including alterations) result in memory cells that are much better able to undergo metabolic reprogramming. CD8^+^ memory T cells, for example, have increased mitochondrial mass and alternate breathing capacity (80, 85), which can be linked to the ability of cells to respond quickly to metabolic increases. Moreover, memory cells selectively express the carrier of Aquaporin-9 glycerol, which is involved in fatty acid storage (86).

The inability to achieve this metabolic change is related with decreased central memory retention or responsiveness (85, 86). Demonstrating that these “fit” memory cells appear to be favoured by mitochondrial remodelling (including networks of fused mitochondria) (78), and subsequent discoveries reveal that CD28 costimulation drives that process (87).

There is much more to be learned about how metabolic pathways regulate survival, differentiation and function in T lymphocytes; for example, the need for FAO in CD8^+^ T_CM_ is satisfied in a way that is difficult to understand: T_CM_ involves importing glucose and glycerol to synthesize and then metabolize triacylglycerides into what appears to be a futile metabolic cycle (i.e. those who consume as much energy as they produce) (88). Synthesis/storage (at times of abundance) and lipolysis (at times of need) ensure the continued survival of T_CM_, but why this is preferred to direct uptake of free fatty acids (FFA) is unclear.

CD4^+^ T lymphocytes also require glucose absorption for sustained maintenance in a pathway controlled by signalling through Notch (89). However, it is unclear how similar metabolic needs are for maintaining CD4^+^ and CD8^+^ T_CM_. The transcription activation of HIF-1α by hypoxia (or other indications) is associated with the promotion of the effector at the expense of the differentiation of memory (90, 91), but may also support epigenetic regulation (92);. Similarly, the regulation of aerobic glycolysis can influence epigenetic transcription and control of gene expression (93, 94).

Interestingly, recent data suggest that there are alternative metabolic strategies for memory T lymphocyte populations. Studies in which CD8^+^ T cells were prevented from switching between aerobic glycolysis and oxidative phosphorylation during an immune response (through sustained HIF activation) showed a surprisingly robust generation of memory cells in lymphoid sites - particularly T_EM_ phenotype cells, suggesting that glycolysis dependency is sufficient to meet the metabolic requirements of at least some CD8^+^ recirculated memory T cells (95).

T_RM_ appears to use another mechanism to treat their metabolic process. As stated above, T_CM_ makes its own fatty acids for stimulating the FAO: the skin, whilst T_RM_, on the other hand, clearly use the more direct route of absorption of free fatty acids (involving the fatty acid-binding proteins Fabp4 and Fabp5) to meet their FAO requirements, as noted by the finding that Fabp4/Fabp5 deficiency selectively affects cutaneous T_RM_ but not the generation of recirculated memory CD8^+^ T cells (45). It will be essential to determine whether this metabolic route is also used in other tissues. These findings illustrate the fact that there are obviously multiple ways to resolve metabolic needs for sustained maintenance of memory cells. It is unclear whether these alternatives will easily correspond to current subset designations.

## General Background of T_SCM_ Cell

While T cell development on the individual cell level is well known, we still lack critical information about how the T cell compartment is formed. Broadly, the T cell compartment is highly heterogeneous and different subsets of T cells based on phenotypical and functional distinct can be identified in the peripheral blood, which is classified, as previously described, as either naïve or memory cells based on their expression (or lack thereof) of various cell-surface markers and receptors. The frequency of memory T cells is highly age-dependent, and it dynamically changes throughout an individual’s lifetime. During the first two decades of life, all T cells in the peripheral blood are naïve. Memory T cells develop over time in response to exposure to diverse antigens, and eventually, circulating memory T cell frequencies reach a plateau and remain stable throughout adulthood (96, 97). Ultimately the proportion and the functionality of memory T cells become altered during immune senescence, starting at 65–70 years of age (97–99).

Recently a subset of memory T cells with stem cell-like properties has been identified with a superior survival potential and stemness function (100). T_SCM_ in humans derived from naive T cells with a multipotent function that can further differentiate into other T cell subsets with a high proliferative capacity and self-renewal flair. Additionally, T_SCM_, defined in humans by surface expression of CD122 (also known as IL−2Rβ), CD95 (also known as FAS), and CXCR3 within the CD45RA^+^CD45RO^–^CCR7^+^CD62L^+^ naïve-like compartment, and they also express high levels of the co−stimulatory receptors CD27 and CD28, IL−7 receptor α−chain (IL−7Rα). These properties define their ‘stem cell-like’ capacities (100, 101).

T_SCM_ cells have been described in peripheral blood and can be either CD4^+^ or CD8^+^. They are a small fraction of circulating T lymphocytes (≈2–3%). Notably, the frequency of circulating T_SCM_ cells can vary because of the different factors that will be explained in this review, but there is evidence to be heritable and associated with single-nucleotide polymorphisms (SNPs) at a genetic locus containing CD95 (102) that showed a potential role of FAS-signalling pathway in the regulation of T_SCM_ cell homeostasis. After many years of tracking the origin of different memory cells to address the stemness function, the studies recently showed that T_SCM_ cells differentiate directly from naive precursors that can reconstitute the entire heterogeneity of memory T cell subsets (31, 32).

## The Role of Metabolic Pathway of T_SCM_ Cells in Infectious Diseases

T cells can be formidable soldiers in the fight against human diseases such as cancer and infection; there is a minority illustrious population due to the capacity to reconstitute a wide-ranging diversity of T cell compartment, robust proliferative potential, and extreme longevity such as T_SCM_, which make them an ideal cell population to use in adoptive immunotherapy or the development of new vaccines against infectious diseases, this might ultimately allow for the widespread application of adoptive immunotherapy based on multipotent, highly proliferative, tumour-specific T_SCM_. Finally, generating long-term T cell memory might be important for T cell-based vaccines designed to target intracellular pathogens, as well as immunotherapy. But we are still in the early stage of this intention because there are many outstanding questions about this very small population, such as their metabolic requirements, epigenetic and transcriptional programs also anatomical niches. So far, the clinical exploitation of T_SCM_ has been limited by their low numbers in the circulation (100, 103) and the lack of robust, clinical-grade manufacturing protocols capable of generating and maintaining this cell type *in vitro*.

Here we will review all the available protocols in detail. In summary, all of these protocols are based on the fate decision that the naïve cell must make to go on a path for proliferation and effector production or memory development.

As explained before, the fate decision that naïve T cells have to make is highly dependent on cellular metabolism or transcriptional modification, but the key question in this critical decision time is how does an activated T cell retain the stem-like capacity to self-renew while simultaneously developing into more differentiated progeny?

**Figure 2:**
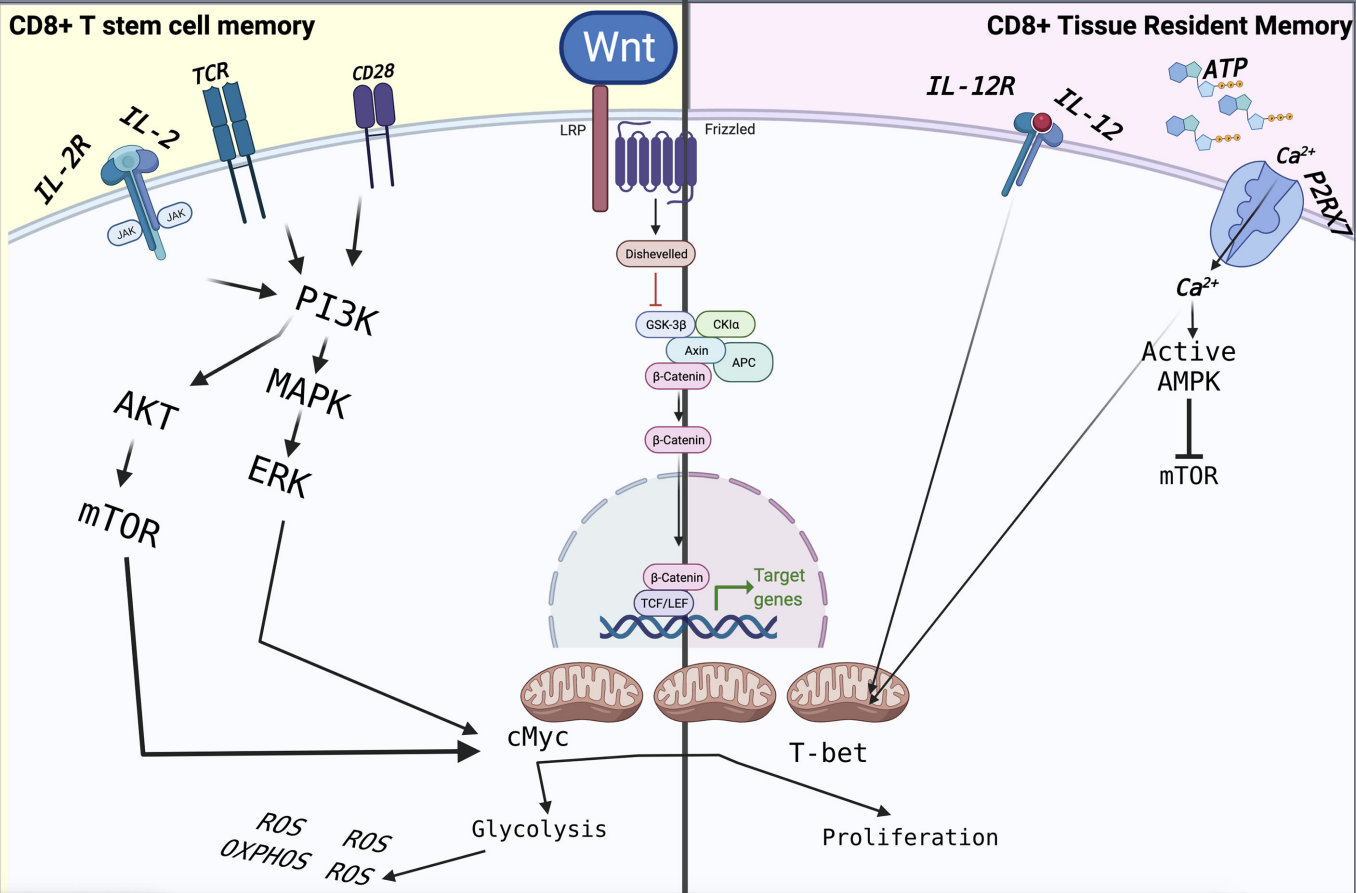
The immunometabolism of T_SCM_ and T_RM_ is driven by extracellular factors, which are mainly based on mitochondrial respiration through FAO for their homeostasis. In particular, the metabolism of TRMs is based on the detection of extracellular ATP mediated by P2XR7, which controls their function and their survival. Following stimulation through TCR and CD28 in the presence of IL-2 and IL-12, CD8^+^ T cells activate reprogramming, differentiating into effector cells that will lead to the elimination of the pathogen or tumour cell. The signalling pathways are those of MAPK and mTOR, which lead to cell proliferation assisted by intense metabolic activity such as glycolysis and OXPHOS. The latter leads to the increased production of ROS, which will induce terminal differentiation and ultimately apoptosis, limiting the overall antitumor effect of CD8^+^ T cells.

Over the last decade, the existed protocol for isolation and *in vitro* expansion of T_SCM_ have been accomplished using modified human T cell expansion protocols by targeting different cellular pathways, such as replacing IL-2 with IL-7 and IL-15, or IL-21 (104, 105), promoting Notch signalling (106) or limiting reactive oxygen species (ROS) metabolism upon antioxidant treatment (106), pharmacological inhibition of GSK-3b (107), AKT (108) or BET proteins (109) also takes to the point as additional strategies, which targeting the suppression of genes associated with terminal effector differentiation, such as Tbet, BATF, and EOMES, and in the maintenance of stem-like genes, such as those encoding TCF1 and lymphoid enhancer binding factor 1 (LEF1) to address the naïve fate decision and force it to generate T_SCM_ (**Figure 2**).

Physiologically, the generation of effector T cells and T cells with stem cell-like properties are linked. In fact, inhibition of lymphocyte differentiation to maintain long-lived, self-renewing antigen-experienced T cells with stem cell-like properties are strictly correlated. A Recent protocol to generate CD8^+^ T_SCM_ is by oxidative metabolism modulation using antioxidants such as N-acetylcysteine (NAC) that significantly increases the intracellular antioxidant molecule Glutathione and consequently decreases the ROS intracellular levels (106). NAC may regulate cellular behaviour in many ways, but the impressive observation is an increased level of Carnitine palmitoyl transferase 1 alfa (CPT1α) and TCF1 expression. From this study, it is now clear that the expression of TCF1 is marked as self-renewing human CD8^+^ T cells (110); furthermore, CPT1 is a transmembrane enzyme of the mitochondrial outer membrane, which converts long-chain fatty acyl-CoA to long-chain acylcarnitine, following which carnitine acylcarnitine translocase, transports the fatty acid across the inner mitochondrial membrane and then enter fatty acid β-oxidation. Therefore, when naïve T cell use *via* s fatty acid for energy *via* OxPhos and decreases dependence on glycolysis, it starts the long-live memory generation. A confirmation for this statement is the study of Seok et al. that demonstrated CPT1 can increase fatty acid oxidation and reduce mitochondrial membrane potential (ΔΨm) (111), which is clearly one of the indispensable characteristics of T_SCM_. The induction of TCF1 and its downstream target ID3 and LEF1 genes are thought to be responsible for the enhanced long-term persistence of stem-like memory T cells. Furthermore, NAC might scavenge ROS levels because it reacts slowly with free radicals such as O_2_^-^, H_2_O_2_ and NO_3_^-^ also, NAC decreases mTOR activity, which it explained as a master regulator of multiple metabolic processes, including glycolysis in T cells. As a result, and according to this explanation, the modulation of mTOR signalling by the simple addition of an inexpensive antioxidant such as NAC in the range of 10–20 mM can triggers the formation of T_SCM_ (112).

An additional strategy in the protocol development to induce *in vitro* cells with T_SCM_ properties is the activation of the Wnt/β-catenin pathway in naïve T cells stimulated through T cell receptor by inhibition of glycogen synthase kinase-3b (Gsk-3b) (107). While the Wnt/β-catenin pathway has been shown to play an essential role in various developmental processes such as asymmetric cell division, stem cell pluripotency, and cell fate specification (113–115), in addition, the Wnt/β-catenin pathway is clearly marked as the essential role of development of T lymphocytes in thymus. Indeed, the role of Wnt proteins in regulating mature T cell function has remained a relatively understudied area. As different Wnt signalling pathways have been described, but our focus is on the canonical Wnt pathway. Actually, Wnt signalling and β-catenin dependent gene expression play a critical role during the formation of different body regions in the early embryo. One of the most important results of Wnt signalling and the high level of β-catenin in certain cell types is the maintenance of stem cell pluripotency (116). Broadly, β-catenin is considered the centre of the Wnt signalling pathway. In addition, the transcriptional activity of the T cell factor (TCF) is induced by direct association with β-catenin (115, 117). However, the expression of β-catenin levels can be carefully modulated by Gsk-3b. The significantly effective way to rapidly accumulate β-catenin to almost 1.7 fold is inhibition of Gsk-3b by a molecule known as TWS119, which initiates the Wnt signalling pathway and controls TCF and LEF transcription factors (115) which acts as the main downstream effectors of the Wnt signalling pathway. The idea behind this protocol is that Wnt signalling functions as a mechanism in T cells to establish a pool of undifferentiated progenitor or stem cells that are more developed than naïve T cells for further differentiation or proliferation. Hence, such applications also require careful study of the regulation of WNT signalling in T cell development and fate. Indeed, small-molecule inhibitors and activators of WNT–β-catenin such as TWS119 are being developed for T_SCM_ generation as therapeutic applications. Primary studies clearly showed that inhibition of GSK3β by 3 μM of TWS119 can arrest CD8^+^ T cell’s development into effector cells, consequently maintaining T_SCM_ phenotype, which can be used as a new vaccination design strategies and adoptive immunotherapies (107). As we explained before, maintaining the stemness function of T cells is dependent on cell metabolism and using more fatty acids as energy *via* OxPhos by decreasing mTOR. Taken together, an existence of cross-talk between mTOR and Wnt signalling comes to mind, which still remains unclear. Furthermore, Scholz et al. revealed that induction of T_SCM_ is utterly independent from the Wnt signalling pathway (118), which has already been called into question as to the molecular mechanism of TWS119, but there is confirming evidence in mouse T cells that TWS119 inhibits mTORC1 (119). In any case, inhibition of mTORC1 can switch the metabolic program of activated naïve T cells toward an oxidative metabolism dependent on FAO, but *in vitro* experiment with TWS119 showed that all naïve T cells do not follow equally this issue (119). A point that needs to be addressed is how a cell’s intrinsic factors can stop differentiation upon mTORC1 inhibition. One of the important clues might be Krüppel-like-factor 2 (KLF2), which has been shown to maintain the expression of CCR7 and CD62L and has been suggested to be up-regulated upon mTORC1 inhibition (85, 120). Interestingly, KLF2 can positively regulate the expression of GSK3β, GATA6 and FOXP3 based on STRING gene interaction data. In addition, the low expression of KLF2 in naive T cells can make them a T_SCM_ cell precursor potential (118).

These two investigations reveal that gene expression differs significantly between *in vitro* these two well-developed *in vitro* induced-T_SCM_ protocols;*in vitro* TWS119-induced T_SCM_ demonstrated an upregulation of LAMP3 (CD63), providing them with a robust co-stimulatory signal, as well as a remarkable elevation of interferon responsive gene expression such as IFIT2, IFIT3, IFI6, and IFI27 Interestingly, mTOR signalling modulated induced-T_SCM_, resulting in the upregulation of genes with cell metabolism control functions such as NQO1, TXNRD1, which can protect cells from oxidative stress and toxic quinones and may be linked to increased oxidative phosphorylation and fatty acid oxidation (118).

## The Role of Long-Term Memory Cells During Chronic Infections

During past years, the role of pathogen-specific T_SCM_ cells in human acute and chronic infections caused by viruses, bacteria, and parasites has been increasingly considered. However, the T_SCM_ development and formation mechanisms during natural immune responses against foreign pathogens remain poorly understood. The critical point of the robust immune response against pathogens is to have effectors not exhausted T cells; this requires continuously replenishment by less differentiated T cell subsets such as T_SCM_ (60, 62, 121, 122). T_SCM_ has recently been linked to a variety of disorders, including autoimmune diseases such as systemic lupus erythematosus (SLE) (123) and infectious diseases (124) and human T cell leukaemia (125). Therefore those subtype of T cells is also taken into account for tumour immunotherapy (126, 127) and allogeneic hematopoietic stem cell transplantation (31, 32).

Longevity and stemness function of T_SCM_ can act as a double-edged sword as protective or pathogenic, as the evidence shows disrupting T_SCM_ cell reservoirs in retroviral infections such as CD4^+^ T_SCM_ or in autoimmune diseases such as aplastic anaemia, rheumatoid arthritis, type I diabetes and SLE, might be a target in diseases controlling strategy. On the other hand, the protective role of T_SCM_ in acute and chronic infections makes them optimal candidates for therapeutic exploitation in vaccination and adoptive T cell therapy against infectious diseases and cancer. One of the best examples of the double-edged role of T_SCM_ in human infectious diseases is controlling HIV or developing AIDS.

However, there is increasing evidence demonstrating that T_SCM_ cells play a crucial role in HIV as a reservoir regardless of successful antiretroviral therapy (ART) (128–131), but immunometabolism data and stem cell-specific pathways can play a very important role in virus resistance since hematopoietic stem cells seem to be largely resistant to HIV-1. Hence, this theory cannot be far from the idea that at least a part of T_SCM_ could have defence mechanisms protecting this cell subset from retroviral infection. A small amount of T_SCM_ showed low expression of CCR5, which can be protected from HIV infection, but physiologically CD4^+^ T_SCM_ expresses CCR5 (R5)- and CXCR4 (X4), which make them usual reservoirs for HIV, because silent or latent infection of these long-lived, self-renewing cells could provide an extremely stable niche for the virus which is modulated by SAMHD1 expression (132). Accordingly, it would need to be targeted T_SCM_ in efforts to eradicate the virus that is appealing for many years after the discovery of T_SCM_ as the stem cells of cellular immune memory from controlling this subset by Wnt-β-catenin to CRISPR-edited stem cells technology (133, 134).

Up to now, the role of HIV-1-specific CD8^+^ T_SCM_ in immune defence is uncertain even if there is evidence that HIV-1-specific T_SCM_ may contribute to HIV-1 restriction and naturally control the infection. Therefore, the proportions of total CD8^+^ T_SCM_ in untreated HIV patients are inversely correlated with plasma viremia levels (135). As mentioned earlier, cell immunometabolism is fundamental for CD8^+^ memory formation. Surprisingly, a recent study found that HIV patients who could naturally control HIV infection had HIV-specific CD8^+^ T imprinted in their memory program, which is associated with their ability to use diverse metabolic resources; in contrast, poor functionality of HIV-specific CD8^+^ was associated with reliance on glycolysis as a primary energy source (136). In addition, this dependency is in contrast whit memory formation with stem cell function and development. Mueller, et al. could convincingly establish that IL15 can enhance the survival and function of HIV specific CD8^+^ T cells by increasing mitochondrial biogenesis (137), which we considered as a fait decision factor for T_SCM_ formation. Taken together, those evidence unveil the importance of metabolic activity regulation and HIV-specific CD8^+^ T_SCM_ formation and consequently induce HIV remission and provide a target for therapeutic vaccine development.

Inducing a long-lasting stem cell-like memory CD8^+^ T cell is not only for protecting human from an infection but also could be a novel target to inducing long-term memory, as yellow fever vaccination evidence shows rapid detection of YF-specific CD8^+^ T cells, but frequency of effector cells decreasing with time while YF-specific CD8^+^ T_SCM_ maintained for more than 25 years and was capable of self-renewal *ex vivo*. Additionally, 17 years after vaccination, T_SCM_ can be prominently distinguished based on their marker maintenance, such as CD58, CD95, CXCR3, KLRG1, CD11a^+^ high, IL-18Rα, granzyme A and IL-2Rβ (138). Accordingly, T_SCM_ thought to be a pivotal T cell subset that have different qualities in terms of effector function, proliferative capacity as well as immune reconstitution and memory formation and it is believed that a T_SCM_ net production can enhance adoptive immunotherapy which even very low numbers of T cells from the memory stem cell pool such as T_CMs_ or T_SCMs_ can reconstitute robust and long term maintained immune responses (11, 139). Given the T_SCM_ role, a major challenge in vaccine development is determining the relative importance of T_SCM_ cells and defining if effective vaccines should induce antigen-specific T_SCM_ for maximal protection.

## T_SCM_ and Autoimmune Diseases

T_SCM_ cells provide long-term protective immunity for anti-tumour immunity, which is likely based on responsiveness to self-antigens; therefore, their involvement in autoimmunity is unavoidable (102, 140). The study of Hosokawa et al. showed that CD8^+^ T_SCM_ cells played an essential role in the pathogenetic mechanism of aplastic anaemia (AA) and were expected to become a new therapeutic direction for this disease (141). An increased CD8^+^ T_SCMs_ frequency at diagnosis is associated with responsiveness to immunosuppressive therapy, and an elevated CD8^+^ T_SCMs_ population after immunosuppressive therapy correlates with treatment failure or relapse in AA patients.

A positive correlation between the frequencies of T_SCM_ CD4^+^ and CD8^+^ cells is seen in AA, autoimmune uveitis and systemic lupus erythematosus (SLE) (141). SLE, a chronic, connective tissue multi-organ disorder that affects young women. Compared to healthy controls, there was a significant increase in the percentage of CD4^+^ and CD8^+^ T_SCM_ cells in SLE patients. T Follicular helper cells (T_FH_) increase antibodies produced by B cells, and T_SCM_ cells play a role in SLE pathogenesis by maintaining T_FH_ cells (142).

Immune thrombocytopenia (ITP), an acquired autoimmune haemorrhagic disease, represents about 30% of all haemorrhagic diseases (143). Many researchers have conducted extensive studies on the pathogenic mechanisms of ITP. At present, there is growing evidence that abnormal cellular immunity plays a significant role in the development of IPT. Very recently, Cao et al., showed that the percentage of CD8^+^ T_SCMs_ in peripheral blood before treatment in ITP patients was significantly higher than that in healthy controls, although some data overlapped in the two groups, whereas the percentages of the other T cell subsets did not exhibit significant differences (143).

Considering that T_SCMs_ can generate all memory and effector T cells, the authors speculated that they could cause the progression of autoimmune diseases. Moreover, the variation in the percentage of T_SCMs_ CD8^+^ is correlated with the effectiveness of the treatment. The percentage of CD8^+^ T_SCM_ in peripheral blood of ITP patients was significantly reduced after glucocorticoid treatment, indicating that the imbalance of the ratio of CD8^+^ T_SCM_ may be involved in the occurrence and development of ITP (143).

Type I diabetes (T1D) results from chronic autoimmune destruction of insulin-producing β-cells mediated by self-reactive T-cells. Many β-cell autoantigens are targets for autoimmunity. Among these, GAD65, (pro)insulin and the block-specific glucose-6-phosphatase subunit catalytic protein (IGRP) appear to be highly antigenic in man (144). The presence of a memory response to islet autoantigens may be related to the limited success of immunotherapies tested to date in T1D patients. Memory autoimmunity is highly resistant to modulation with immunosuppressant drugs and immunomodulatory molecules (145, 146), primarily designed to target activated and fast-proliferating T-effector cells. These raised the question of whether self-reactive and long-lasting T_SCM_ precursors can serve as a reservoir of self-reactive clones in post-treatment autoimmune relapses (147). Identifying specific circulating T_SCM_ for GAD65, insulin, and IGRP supports this assumption and provides a new target cell population to design novel immunotherapy approaches. Selective targeting of self-reactive T_SCM_ clones may be used to suppress more differentiated T-cells permanently.

Further efforts are needed to clarify changes in health and disease stages among the different T_SCM_ cell states.

## General Background of T_RM_ Cells

T_RM_ cells are an essential part of the immune survey system, sensing alteration in homeostasis throughout the body and leading immune surveillance of most non-lymphoid tissues (148–151). T_RM_ cells are involved in protection from infection and cancer, but also likely promoting autoimmunity, allergy, and inflammatory diseases and impede successful transplantation. The residence signature that marks T_RM_ cells in several tissues is characterized by both increased expression of proteins that promote tissue retention and reduced expression of proteins involved in the recirculation in the blood and lymphatic vessels.

For instance, T_RM_ cells exhibit reduced expression of the surface molecules such as S1PR1 and CCR7 cells, which promote T cells to leave the NLT, an observation that is explained by a decreased expression of the transcription factor KLF2, which leads to transcription of S1PR1 and CCR7 (152). On the other hand, T_RM_ cells express CD69 and, in the case of T_RM_ cells located in epithelial tissues, the E-cadherin αE integrin binding (CD103, encoded by ITGAE gene), which both promote tissue retention (29).

T_RM_ cells as all the memory T cells express CD44 and lack CD62L, while T_RM_ cells are exclusively CD44^hi^ and CD62L^low^ they can display several tissue-specific markers, so that no single marker can unique identify a T_RM_ population in all tissues.

Thus, the expression of CD69 and CD103 should be considered an imperfect marker for inferring tissue residence because the absence of their expression does not exclude the potential to leave NLTs (151, 153–156).

CD103, CD49a (VLA-1) and CD69 are the most commonly utilized markers to distinguish T_RM_ cells from circulating memory cells (157). CD103 is a ligand for E-cadherin, widely expressed in the junctions between epithelial cells at barrier sites (158). CD49a is an integrin ligand for collagen IV which is located in the lamina dense of barriers tissues (159). Moreover, CD49a has an anti-apoptotic function for the collagen bounding T lymphocytes (160). CD69 is an S1P1 antagonist, countering signals for lymphocyte egression and circulation (161, 162). CD69 is expressed by the majority of T_RM_, CD103 is mainly expressed primarily by CD8^+^ T_RM_ cells (163). Many other cell surface markers are associated with T_RM_ cells, including CXCR6, CD101, PD-1, and CX3CR1.

Nevertheless, a large part of our current understanding of T_RM_ cells relies on analyses of CD69^+^CD103^+^ T_RM_ cells in epithelial tissues.

## Role of T_RM_ in Chronic Infectious Diseases

Consistent with their role as local sentinels, it has been demonstrated that T_RM_ cells both prevent and exacerbate pathologies. For example, T_RM_ cells protect local recurrent pathogens (148, 164) but these cells can also provide protection against the development of skin malignancies (165–167).

T_RM_ cells can lead to immunotherapy-induced colitis (168), autoimmune disorders of the skin vitiligo (169) and psoriasis (170, 171) as well as other autoimmune and allergic diseases, and can play a central role in allograft rejection (172).

T_RM_ constantly perform surveillance of the cells surrounding them and intimately interact with the components of their environment including epithelial cells and the extracellular matrix. For this reason, T_RM_ are among the first line of defence and are early activated by the encountering of antigens. The involvement of T_RM_ cells in a series of human diseases makes the design of therapeutic strategies that can modulate both their production and their activity an attractive objective, and to realize this goal, understanding how this cell pool is formed is essential (173).

While studies on the role of T_RMs_ in cancer are very extensive, the role of T_RMs_ in the context of chronic infections is less explored. In general, there is strong evidence that chronic tissue-specific infection is associated with a non-functional alteration of the T_RM_ population evidenced by an exhausted phenotype. However, even in this context some studies have highlighted the possibility of using this subset for vaccination or therapeutic purposes against infectious diseases such as HIV and tuberculosis.

For instance, a recent study showed that in HIV patients, CD8^+^ T cells HIV specific with a T_RM_ phenotype are abundant in HIV infected lymph nodes compared to peripheral blood. These CD8^+^ T_RM_ cells show different specificity toward different HIV antigens, and this finding results in a different clonotype observed in different HIV infected lymph nodes (155).

The presence of HIV specific T_RM_ cells in lymph nodes of HIV infected subjects makes these cells suitable targets of a vaccine against HIV. A recent study on murine model based on subcutaneous microneedles delivery array (MA) vaccination with recombinant E1, E3 deleted human adenovirus type 5 (AdHu5) vector encoding HIV-1 CN54gag (AdHu5-CN54gag), induced in the skin the a subset of specific memory T CD8^+^ cells with a T_RM_ phenotype (CXCR3^+^, CD103^+^, CD49a^+^, CD69^+^) and a polyfunctional profile (174).

In a recent study on the tuberculosis pleural effusion (TPE), the authors found a heterogeneous population of T cells in which CD103^+^CD8^+^ T cells were the predominant subset of CD103^+^ lymphocytes. Moreover, the frequency of CD103^+^ CD69^+^ memory CD8^+^ T cells in TPE was significantly higher than the frequency of these cells in peripheral blood. Altogether, CD103 and CD69 expressions define in TPE distinct CD8^+^ T_RM_ -like subsets with different phenotypes and functions (175).

The consistent presence of T_RM_ in lungs focused the attention of many scientists on these cells as a target for vaccine or host direct therapy against *Mycobacterium tuberculosis* infection (Mtb).

Moreover, different studies on possible mucosal TB vaccines against TB have been carried out on mouse models, revealing the marked presence of both CD8^+^ and CD4^+^ T_RM_ in the lungs. In a study on murine models, the intranasal boosting with a Sendai virus vectored tuberculosis vaccine, SeV85AB, induced antigen-specific CD103^+^CD8^+^ T-cell protective response in the lung, associated with a reduction of mycobacterial burden both in lung and spleen compared to subcutaneous BCG alone vaccination (176). Nanoparticles (NPs) are an emerging vaccine technology, with recent oncology and infectious diseases successes. Hart et al., have associated Mtb control by NPs due to the enhanced cellular immunity compared to BCG, including superior CD4^+^ and CD8^+^ T cell proliferation, T_RM_ seeding in the lungs, and cytokine polyfunctionality (177). In another study, *Bacillus subtilis* spores coated with a fusion protein 1 (“FP1”) consisting of Mtb antigens Ag85B, ACR, and HBHA have been tested in a murine low-dose Mtb aerosol challenge model (178). This vaccination model demonstrated an enhanced pulmonary control of Mtb, as evidenced by reduced bacterial burdens in the lungs. Spore-FP1 immunization generated superior antigen-specific memory T-cell proliferation in both CD4^+^ and CD8^+^ compartments. Moreover, CD69^+^CD103^+^ T_RM_ cells were found within the lung parenchyma after mucosal immunization, confirming the advantages of mucosal delivery (178). Finally, liposome-based subunit vaccine formulation of phosphatidylserine encapsulating, Mtb antigens Ag85B, and ESAT-6, given mucosally, could generate T_RM_ cells, that have been found in the lung parenchyma that are likely to be antigen specific, even if no formal evidence of their antigen specificity was demonstrated (179).

The administration of BCG by intranasal rote elicited the generation of both CD8^+^ CD69^+^CD103^+^ CXCR3^+^ T_RMs_, able to improve the protection against Mtb challenge when compared to the simple administration of subcutaneous BCG (180).

Adoptive transfer of these cells to naive, TCRα^−/−^ transgenic mice, prior to Mtb infection, demonstrated parenchymal T-cells, which expressed the T_RM_ CXCR3^+^KLRG1^−^ phenotype, had far greater activity than circulating T-cells (18-fold vs 4-fold reduction in lung bacterial burden) (181).

An interesting finding regarding T_RM_ cells in the tuberculosis murine model is the expression of PD-1, which is a key molecule to modulate the IFN-γ production and thus, prevent lethal lung disease. This finding was assessed by a recent study showing the generation of protective KLRG1^−^PD1^+^ T_RM_ cells in lung after mucosal vaccination with BCG, but not after intradermal BCG administration (182).

## T_RM_ Generation and Homeostasis in the Barrier and Mucosal Tissues

T_RM_ cells are a dedicated population of T cells that continuously recirculate through tissues or whether they can permanently reside and show decisive functions, organizing local protective immune responses. This migration, a feature of classical memory T cell subsets, is the basis of the differential expression of CCR7 (22). Once the pathogen is encountered, it expands in situ, acquiring marked cytotoxic activities, promoting the formation of a tissue antiviral environment and releasing numerous cytokines (183, 184). Indeed, T_RM_ cells’ maturation foresees the encounter with the antigen at the tissue level resulting in the downregulation of CCR7 in the activated cells, and this process is a prerequisite for the formation of T_RM_ (185–187). Furthermore, a study on a mouse model, based on the intranasal or intramuscular administration of CpG and nucleoprotein peptides (NP) from influenza A/HKx31 (H3N2) virus, showed the formation of NP-specific T_RM_ in the respiratory tract tissues only in mice that had had intranasal administration of the NP (188).

Those functions generally require fundamental immunometabolism changes, metabolic homoeostasis, and remodelling in T cells during life and ageing. All of this remains unexplored, but it is now clear that during humans ageing, progressive deterioration leads to impaired function and continual decline that results in an increased risk of disease and death (189); as an example, a recent study clearly shows how over the years there is a variation in the passage of immune cells in the lungs and how these cells respond specifically against classic influenza and SARS-CoV-2 virus (190). In fact, most immune cells in human lungs remain stable with age, unlike memory CD8^+^ T cells that decrease, particularly the T_RM_ cells (191, 192). As seen in Nguyen et al. studies, a subgroup of CD103^+^ and CD69^+^ T_RM_ cells in the lungs of donors under the age of 50 accounts for about 48% of the total memory CD8^+^ T cells, but in subjects over 50 years of age, it represents only 25% of CD8^+^ memory T cells (190).

These observations can be realized through ageing and T cell immunometabolism; for example, ageing is associated with reduced TCR diversity (193) and shifting from naïve to memory subset, and these phenotypic changes result in an overall compromised response to new antigens. An example to prove this fact is that the overall frequency of influenza-specific CD8^+^ pulmonary memory T lymphocytes does not show variations with the donor’s age, while the subgroup of influenza-specific T_RM_ lymphocytes with phenotype CD103^+^, CD69^+^ decreases with age. This decrease coincides with the reciprocal increase in the CD103^-^ and CD69^-^ population of CD8^+^ T_RM_ T lymphocytes, influencing the quality of the early inflammatory response following exposure to the virus (190).

Homoeostasis and remodelling in T cells during life and ageing leads to a marked susceptibility of the elderly to the most serious forms of virus infection. For example, in influenza, specific CD8^+^ T_RM_ T cells in the lung rapidly acquire proinflammatory effector functions directly in situ, thus being decisive in fighting a viral infection (194, 195).

## The Role of Metabolic Pathway of T_RM_ in Infectious Diseases

Barrier tissues and their microenvironments have different trophic and pH conditions that T_RM_ cells should face. Moreover, the cell populations, the extracellular matrix, and the collagen type are different. For this reason, T_RM_ cells can exploit diverse sources of energy by different metabolic pathways.

Metabolic reprogramming and metabolism changes are associated with adapting specific T cell subunits for survival and cell function, supporting cell function through energy supply and biosynthetic precursors. For example, cellular and transcriptional changes during an infection are responsible for developing different memory CD8^+^ T cells and antigen-specific CD8^+^ T cells. In addition, many changes can control CD8^+^ T cell metabolism in response to infection, driven by extracellular metabolites, such as transcriptional, translational, and epigenetic changes.

During acute infection, one of the most abundant CD8^+^ T_RM_ cells become embedded in lymphoid and non-lymphoid peripheral tissues (151, 196). However, after pathogen clearance, only a few T cells remain as long-lived memory CD8^+^ T cells such as T_RM_ or T_SCM_, which are critically dependent on cellular metabolism to regulate CD8^+^ T cell differentiation and memory formation (197–200). For example, an essential requirement for the differentiation of T_RM_ cells and T_CM_ CD8^+^ T cells is purinergic receptor P2RX7 (201), which polymorphism on this gene plays a role in susceptibility to TB in the Asian population (202). In fact, non-lethal P2XR7 maintains mitochondrial homeostasis and TGF-β dependent programs of tissue residency by increasing AMPK activation compared with other tissue CD8^+^ T cells. CD8^+^ T_RM_ appears to possess a distinct functional and metabolic profile that can depend on their environment and adapt to it. CD8^+^ T_RM_ maintains long-term survival by using FAs which closely depend on extracellular factors such as extracellular ATP, which could influence the T_RM_ metabolism and expression of CD69 and CD103 (201).

As an example, P2XR7 can affect the mTOR pathway to switching from glycolysis to OXPHOS (203). Unlike circulating memory CD8^+^ T that mainly depends on glycolysis to respond to pathogenic antigen (80), T_RM_ depends on the uptake of exogenous FAs for subsequent FAO engagement and subsequently mitochondrial membrane potential and fatty acid metabolism for their homeostasis. Many microenvironment factors such as FA-rich sites, extracellular ATP (eATP) and many receptors can affect gene upregulation which eventually changes effector function and lipid metabolism on T_RM_. For example, the low-affinity P2RX7 receptor to eATP promotes the NLRP3 inflammasome activation (204). In contrast, P2RX7 can promote CD8^^+^^ T_RM_ cell subsets through mitochondrial health (205) (**Figure 2**). Moreover, due to increased infected cell death or gene expression upregulation to change effector function in infection sites, the extracellular NAD^+^ concentration increases, promoting CD8^+^ T_RM_ cell death by P2RX7 (206).

Another pathway used by T_RMs_ exploits free fatty acid (FFA). T_RM_ cells showed a high expression of the fatty acid-binding receptors FABP4, FABP5, low-density lipid receptor, ApoE, and the CD36 scavenger receptor. These molecules are useful to enhance lipolysis for rapid exploiting the direct pathway of FFA uptake (45). The FFA can be used in oxidative phosphorylation and in FAO (45, 207). Thus, it is likely that T_RM_ can switch their metabolic pathway from oxidative phosphorylation and glycolysis to FAO after antigen encounter and subsequent activation.

The master transcriptional regulators of T_RM_ cells differentiation in different tissues are Hobit, Blimp1 (163), and Runx3 (208) at the transcriptomic level. The first two transcriptional factors are negative regulators of tissue egresses (209) and are involved in the T_RM_ cells cytotoxic capability (210). Runx3 enhances the expression of CD103 and CD69 tissue retention molecules (208).

In conclusion, the fate decision by metabolism change in T_RM_ cells remains largely unknown. Those cells could be a fascinating target not only for vaccination efficiency or controlling infection but also in tumour and autoimmune tissue disorders due to the double-edged sword character. Given this content, understanding in detail metabolic reprogramming to possible manipulation to enhance or decrease T_RM_ longevity and function is crucial.

## T_RM_ Cells and Autoimmune Disease

T_RM_ cells have been shown to play a role in autoimmune diseases, especially autoimmune skin diseases. In vitiligo, it showed the presence of T_RM_ cells in the epidermis and dermis of lesioned areas. These cells expressed CXCR3, whose binding with its ligands CXCL9 and CXCL10 drive the production of IFN-γ, which represents an effector cytokine of this pathology. Moreover, these T_RM_ cells produced high quantities of IFN-γ and GRZB after *in vitro* stimulation (211). The phenotype of vitiligo associated with T_RM_ cells also displays the α chain of IL- 15 receptor and CD122, which play an essential role in the pathological activity of T_RM_; in fact, the blocking of CD122 by monoclonal antibody leads to the repigmentation of vitiligo areas in an experimental mouse model (169).

Like vitiligo, psoriasis is an autoimmune skin disease, where IL-17 sustains its pathogenesis. CD8^+^ T_RM_ cells from psoriatic lesions showed IL-17 production after *in vitro* restimulation. These cells display a specific phenotype (CD103^+^ CD49a^-^ CCR6^+^) characterized by the expression of IL23R and the absence of CXCR3 (211, 212). Several studies reported the involvement of T_RM_ cells in other autoimmune diseases. CD8^+^ T_RM_ cells specific for astrocytes-expressed protein play a crucial role in multiple sclerosis (MS) pathogenesis in a mouse model (213, 214). Moreover, CD103^+^ cells were detected in the high inflammatory infiltrate in the lesioned central nervous system (CNS) zones of MS patients (215). But their role remains controversial as these cells, even if stimulated, do not produce cytokines (214). Another study reported the presence of IFN-γ, IL-18, and IL-22 producing CD8^+^ cells in lesioned Langerhans islet in TD1 patients (216).

Several factors should be considered about the mechanisms that trigger the pathological activation of CD8^+^ T_RM_ cells in the tissues associated with autoimmune disease. For skin diseases, keratinocytes, dendritic cells, and fibroblasts, after stimulation, can determine the production of IL-7, IL-15, and IL-17 that can trigger CD8^+^ T_RM_ cells’ activation and enhance their proliferation and the maintenance of a stable pool of activated cells (183). CD8^+^ T_RM_ cells in response to IL-7 and IL-15 can rapidly be activated by the JACK/STAT5 pathway, which profoundly influences the formation of long-lived memory T cells to enhance the anti-apoptotic BCL-2 molecule (217–219). Also, the PI3K/AKT pathway activation is associated with long-lived memory T cells (217, 220, 221). An appropriate balance of the signals from these two pathways is critical either in activated CD8^+^ T_RM_ cells or their survival (222).

It was demonstrated that cross-talk between T_reg_ and T_RM_ cells is linked to the ability of T_reg_ to make TGF-β available for the T_RM_ formation and corroborated by similar expression of CXCR3, which allows both cell types to migrate toward the site of infection and inflammation (223, 224). The role of T_reg_ can prevent pathological activation of T_RM_ cells. Nevertheless, it happens that impaired function of T_reg_ in the skin leads to the formation of T_RM_ cells with autoimmune activity. To confirm this hypothesis, it was observed that the checkpoint inhibitor therapy used in patients with melanoma is associated with an increase in vitiligo disease (169, 225).

About metabolic pathways used by T_RM_ cells during their activation in autoimmune diseases, skin T_RM_ cells can exploit FFA and mitochondrial oxidative metabolism. Together with the fact that skin autoimmune disorders are often concomitant with metabolic diseases, these findings suggest that the metabolic disorders determine a more likely uncontrolled activation of CD8^+^ T_RM_ cells due to an enhanced FFA metabolism. Evident lipid accumulation in droplets (45) and high expression of fatty-acid binding proteins (FABPs) in T_RM_ cells (45, 226) determine the higher expression of transcripts of enzymes involved in lipid metabolism, which supports this hypothesis. In conclusion, the presence of dysfunctional CD8^+^ T_RM_ cells in lesions caused by autoimmune diseases makes the T_RM_ cells subset an important player in the onset and chronic assessment of these diseases and, at the same time, a promising target for new therapeutic strategies in autoimmune diseases.

## T Cell Memory Subsets in SARS-COV-2 Infection or Vaccination

Within the T cell memory compartment, T_RM_ and T_SCM_ CD8^+^ T cells play a different role in SARS-COV-2 infection or vaccination. The first subset has been detected in the respiratory tract following coronavirus infection, where they employ a protective function (227). Moreover, activated profiles of lung CD8^+^ and CD4^+^ T_RM_ cells were reported in COVID-19 patients, and these cells were frequently detected even ten months after infection (228, 229). Recovered patients acquire T_RM_ cells with Th1 phenotype against COVID-19 (229). It was shown that, although resident T cells are elicited by COVID-19 infection, they do not present sufficient protection against secondary viral infection (230). On the other side, CD8^+^ T_RM_ cells can significantly contribute to lung damage/pathology development, determined by the prolonged activities after respiratory viral infection (231). In fact, lung CD103^−^ CD69^+^ T cells of older patients with COVID-19 produce elevated inflammatory molecules associated with worse lung pathology and reduced lung function (232). It has been found that respiratory CD103^-^ CD69^+^ T cells exacerbate lung pathology and reduce lung function (232). Therefore, several therapeutic strategies have been developed to target the T_RM_ cells. A recent study has evaluated the role of Tofacitinib (the FDA approved oral JAK2/1/3 inhibitor) targeting the tissue memory CD8^+^ T cells, which may be an effective therapy against chronic lung diseases promoted by acute SARS-CoV-2 infection (233). SARS-CoV-2 specific memory T_SCM_ cell responses are induced after recovery from SARS-CoV-2 infection or vaccination, according to increasing data. After ten months of infection, Jung et al. discovered a successful generation of T_SCM_ cells in convalescent COVID-19 patients (234). Another study clearly showed that an antigen-specific population of T_SCM_ is observed even after six months of vaccination with BNT162b2 vaccine, which provides a multipotent memory reservoir (235). It was recently revealed that stimulation of T cells shortly after BNT162b2 vaccination with spike epitopes leads to rapid production of TNF-α, which was independent of the neutralizing antibodies’ titer (236). The production of TNF-α, even within a few hours after TCR-stimulation, is most characteristic of naive-like CD8^+^ T cells, similar to T_SCM_ (237). The high production of TNF-α is not always related to a good immunological response, due to its involvement in several inflammatory diseases or the severity of long COVID patients (238–240). In conclusion, T_RM_ and T_SCM_ cells represent a double-edge sward in SARS-Cov-2 infection, and further studies are needed in order to delineate their role better.

## Concluded Remarks

The recent application of high-level single-cell technologies and epigenetic profiling approaches has revealed that the stem and the effector properties of CD8^+^ T cells are no longer reciprocal. Transcriptional programming that equips a T cell with a multipotent development capability can coexist with effector programs and establishes a population of long-lived memory T cells that can respond against a previously exposed pathogen. Recent studies investigating these properties in T cell responses to chronic antigen sources illustrate an essential role of these coexisting characteristics in establishing therapeutic modalities based on selected subsets of T lymphocytes. The great interest in studying the impact of the metabolic activity of pathways in immune cells has opened a new window in understanding the requirements of metabolic pathways for differentiation/maintenance of CD8^+^ T cells, which can lead to the discovery of molecular targets capable of developing new anti-infective and/or anti-inflammatory drugs applicable to different contexts (241–244).

The outstanding issues surrounding these fundamental properties of T cells are of great translational importance and represent a question of intense investigation. These processes will likely provide more information on the cellular and molecular mechanisms of memory T-cell differentiation and exhaustion in acute and chronic pathogenic challenges, allowing the development of new therapeutic approaches that use the exquisite specificity and longevity of T cells.

## Author Contributions

Conceptualization and Supervision, FD, NC; Writing-Review & Editing, BT MPLM, MSA, LM, GDB; Writing-Original Draft Preparation, NC, FD, MPLM; BT, MSA, LM; Figure preparation: MPLM, BT, GDB, MSA, LM. All authors have read and agreed to the submitted version of the manuscript.

## Funding

This work was supported by grants from the Horizon n2020 Programmes EMI-TB (contract no. 643558). The text represents the authors’ views and does not necessarily represents position of the European Commission, which will not be liable for the use made of such information.

## Conflict of Interest

The authors declare that the research was conducted in the absence of any commercial or financial relationships that could be construed as a potential conflict of interest.

## Publisher’s Note

All claims expressed in this article are solely those of the authors and do not necessarily represent those of their affiliated organizations, or those of the publisher, the editors and the reviewers. Any product that may be evaluated in this article, or claim that may be made by its manufacturer, is not guaranteed or endorsed by the publisher.
